# Combination of Diet Quality Score, Plasma Carotenoids, and Lipid Peroxidation to Monitor Oxidative Stress

**DOI:** 10.1155/2018/8601028

**Published:** 2018-12-31

**Authors:** Yunsoo Kim, You Jin Kim, Yeni Lim, Bumjo Oh, Ji Yeon Kim, Jildau Bouwman, Oran Kwon

**Affiliations:** ^1^Department of Nutritional Science and Food Management, Ewha Womans University, Seoul, Republic of Korea; ^2^Department of Family Medicine, Seoul Metropolitan Government-Seoul National University Boramae Medical Center, Seoul, Republic of Korea; ^3^Department of Food Science and Technology, Seoul National University of Science and Technology, Seoul, Republic of Korea; ^4^Microbiology and Systems Biology Group, Netherlands Organization for Applied Scientific Research (TNO), Zeist, Netherlands

## Abstract

It is important to understand the association between oxidative stress-related parameters and to evaluate their status in advance of chronic disease development. Further development towards disease can then be prevented by dietary antioxidants. The present study was aimed at assessing the relationship between diet quality, blood antioxidants, and oxidative damage to determine whether the association between these markers differs by oxidative stress status. For a cross-sectional analysis, we used data and samples of baseline information from a prospective cohort study. A total of 1229 eligible adults were classified into apparently healthy subjects (66.5%) and those with oxidative stress conditions (35.5%). Diet quality was assessed using the recommended food score (RFS). Plasma carotenoids (blood antioxidants) and blood/urinary malondialdehyde (MDA; oxidative damage) were determined by high-performance liquid chromatography. We found that the healthy group was younger, and they had a lower RFS and plasma MDA level and higher plasma carotenoids compared to the oxidative stress condition group. This result is probably due to the quenching of the oxidative response in the tissues of those people. A positive association of RFS with plasma carotenoids (total and *β*-carotene) was found in both groups, suggesting that carotenoids are a robust reflection of diet quality. Negative associations were observed between plasma MDA and RFS in the oxidative stress condition group and between urinary MDA and plasma zeaxanthin in the healthy group. Erythrocyte MDA was positively associated with plasma carotenoids (total, lutein, zeaxanthin, *β*-cryptoxanthin, and *α*- and *β*-carotene), regardless of health condition, probably also as a result of the use of carotenoids as antioxidants. In conclusion, these results indicate that the above three factors may be associated with the oxidative stress response and depend on the oxidative status. Furthermore, it was also suggested that erythrocytes are important in the oxidative stress response and the quenching of this response is represented in plasma carotenoids.

## 1. Introduction

Oxidative stress can be defined as the imbalance between the concentrations of reactive oxygen and nitrogen species (ROS/RNS) and the antioxidant defense mechanism of the body [1]. Chronic oxidative stress has a strong association with certain health conditions, such as metabolic syndrome [1], obesity [2], and diseases, including type 2 diabetes [3], cardiovascular disease [4], and diet-related cancers [5, 6]. Studies reported that consumption of abundant antioxidant foods is connected to a reduction in the risk of oxidative stress-related disease [7, 8]. Moreover, low concentrations of carotenoids have been shown in subjects with oxidative stress-related diseases [9, 10]. It is thus crucial to evaluate the possible role of oxidative stress in the complex pathophysiology of its related diseases at an early stage, to prevent the development of diseases, by nutritional intervention.

It is becoming more pertinent that the conduct of nutrition research requires meaningful composite biomarkers, which may provide a more effective means for monitoring disease progression and response to intervention than the individual biomarkers [11]. These biomarkers may include dietary exposure, nutritional status, or physiological function, which, in turn, influence health and disease risk [12]. A state of oxidative stress can be measured through increased production of prooxidant compounds and depletion of antioxidants [13]. The recommended food score (RFS) is a useful marker of antioxidant food consumption, plasma carotenoids are a useful marker for dietary exposure and plasma antioxidant status, and plasma and urine malondialdehyde (MDA) are a useful marker for oxidative damage [14]. The RFS, derived from a questionnaire, is a diet quality score constructed by summing the consumption of antioxidant-rich foods [15]. In the present study, we focused on the antioxidant carotenoids, including lutein, zeaxanthin, *β*-cryptoxanthin, and *α*- and *β*-carotene. As these cannot be synthesized by humans, circulating carotenoids are reliable biomarkers to determine the exposure of the dietary antioxidants from fruits and vegetables [16]. When there is either an increase in exposure to ROS/RNS or a decrease in antioxidant levels, oxidative damage may occur in lipids, proteins, or DNA [17].

This study was aimed at assessing the cross-sectional associations between RFS, plasma carotenoids, and MDA (plasma, erythrocytes, and urine) to determine whether the association between these markers differs according to the health condition in an East Asian population. Accordingly, the association between RFS and plasma carotenoids was examined to see whether plasma carotenoids can be used as biomarkers for dietary antioxidant intakes. Furthermore, we also examined the extent to which RFS and circulating carotenoid concentrations are associated with MDA levels in plasma and erythrocytes. We used baseline data and samples of Korean adult men and women from a prospective cohort study. The concentrations of carotenoids and MDA in plasma, erythrocyte, and urine samples were analyzed by high-performance liquid chromatography (HPLC). Health condition was divided into two groups: apparently healthy subjects and those with oxidative stress conditions.

## 2. Materials and Methods

### 2.1. Study Design and Subjects

The Ewha–Boramae study is a prospective cohort study of Korean men and women aged 19 yr or above, who underwent a comprehensive annual or biennial health examination. Participants were recruited at Seoul National University Boramae Hospital (Seoul, South Korea). Analysis of carotenoids and MDA in biological samples was conducted at Ewha Womans University (Seoul, South Korea). About 86% of the participants were recruited from those who received a biannual free-of-charge health examination covered by the Framework Act on Health Examination, while the remaining participants were from those who voluntarily participated in a noninsured private health examination.

The cross-sectional analyses in this study were conducted with baseline data from 1464 participants aged 19–80 yr, which were collected between April 2015 and August 2016. The presence of oxidative stress conditions was ascertained from self-reported information on previous diagnoses or use of medication. The terminology of diseases was adopted from the Human Disease Ontology BioPortal (http://purl.bioontology.org/ontology/DOID), which is a comprehensive hierarchical controlled vocabulary for enabling interoperability between biological and clinical human disease descriptors. Oxidative stress conditions were defined as the presence of metabolic syndrome [1], obesity [2], or diseases, including type 2 diabetes [3, 18], hypertension [19], dyslipidemia [19], cardiovascular/neurovascular diseases [18, 20, 21], or diet-related cancers (liver, colon, stomach, breast, prostate, and lung) [5, 18, 22].

**Figure 1:**
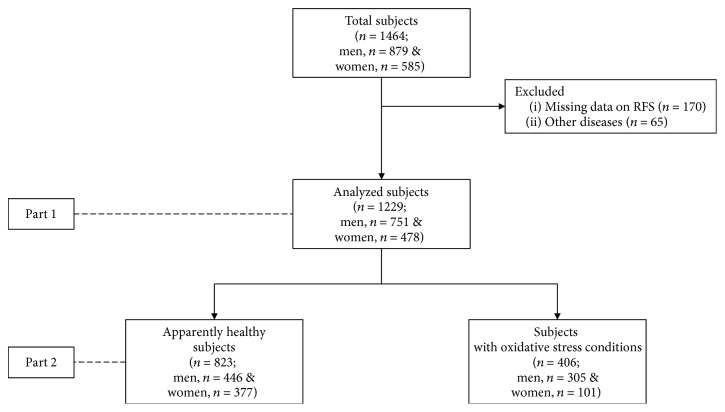
Flowchart of participants recruited in the study.

Of a total 1464 participants who completed a health examination and self-administered questionnaire, we excluded participants who had incomplete data of RFS (*n* = 170) and other diseases besides oxidative stress-related disease (*n* = 65). Therefore, the 1229 eligible participants (*n* = 751 men and *n* = 478 women) were divided into two groups, as apparently healthy subjects (*n* = 823) and subjects with oxidative stress-related disease (*n* = 406) (Figure 1).

This study was approved by the Institutional Review Board of Boramae Hospital (Approval number: 20140929/26-2014-118/102) and Ewha Womans University (Approval number: 86-8). Written informed consent was obtained from all participants after a full explanation of the procedures.

### 2.2. Anthropometric and Metabolic Measurements

Height and weight were recorded to the nearest 0.1 kg and 0.1 cm. A tetrapolar 8-point tactile electrode system (InBody 3.0, Biospace, Seoul, Korea) was used to measure the body weight, body mass index (BMI), and body fat percentage. Waist and hip circumference were measured according to the World Health Organization (WHO) guideline [23]. Systolic and diastolic blood pressures were measured using an automated sphygmomanometer on the right arm in sitting position.

Information on demographic characteristics, smoking status, physical activity, medical history, and medication use was collected through self-administered questionnaires. Smoking status was categorized into never, past (defined as participants who quit over a year ago), or current smoker (defined as those who smoked at least 100 cigarettes in their life). The short version of international physical activity questionnaire (IPAQ) [24] was used to assess physical activity in the past 7 days, from which the relative energy expenditure of each activity was calculated by multiplying weekly minutes of activities with the accompanying the metabolic equivalent task score from the IPAQ protocol [25].

Venous blood and urine samples were collected after 12 hr overnight fasting state in the early morning. Whole blood was collected into an ethylenediaminetetraacetic acid-containing tube, and plasma was obtained immediately by centrifugation at 1600 ×g for 15 min at 4°C. Erythrocytes were washed, subsequently resuspended in distilled water to adjust hematocrit at 25% [26], and hemolyzed by isotonic solution (0.85% sodium chloride). Total cholesterol, triglycerides, low-density lipoprotein cholesterol, high-density lipoprotein cholesterol, and glucose were determined using an automated hematology analyzer (Sysmex XE-2100, Angers, France). For measurement of carotenoid and MDA levels, biological samples (plasma, urine, and erythrocytes) were transported to Ewha Womans University and stored at −80°C until analyzed.

### 2.3. RFS

An RFS was developed by Kant et al. [27] and modified as appropriate to the Korean diet by Kim et al. [15] to evaluate overall diet quality by summing the number of antioxidant-rich foods recommended by dietary guidelines. A total of 46 foods or food groups corresponding to recommended food groups were selected, and one response for “daily frequency of meals” was used to calculate the RFS. Thus, the available maximum score would be 47. Participants received 1 point if they consumed the recommended food or food groups at least once a week or if they ate three meals daily on a regular basis. The selected food or food groups for the RFS were as follows: grains (1), legumes (4), vegetables (17), seaweeds (2), fruits (12), fish (5), dairy products (3), nuts (1), and tea (1).

### 2.4. Plasma Carotenoids

Plasma carotenoid levels were determined by HPLC (Shiseido Co. Ltd., Tokyo, Japan) with a photodiode array (PDA; Shiseido Co. Ltd., Tokyo, Japan) and a YMC C30 column (5 *μ*M, 4.6 × 250 mm; YMC Europe GMB, Dinslaken, Germany). We chose to measure lutein, zeaxanthin, *β*-cryptoxanthin, *α*-carotene, and *β*-carotene because they are all carotenoids that act as antioxidants [28]. The mobile phase consisted of a mixture of solvents A (methanol : methyl *tert*-butyl ether = 95 : 5) and B (methanol : methyl *tert*-butyl ether : 1% ammonium acetate = 8 : 90 : 2), with the following gradient program: 95% A from the beginning of the run to 7.9 min; 90% A from 8.0 to 16.9 min; 55% A from 17.0 to 19.9 min; 42.5% A from 20.0 to 21.9 min; and 90% A from 22.0 to 24.9 min, followed by an equilibration at initial conditions for 5 min. The mobile phase flow rate was 1 mL/min, and the total run time was 30.0 min. Chromatograms were monitored at 221 and 450 nm.

### 2.5. MDA in Biological Samples

The optimized and validated HPLC analytical method was applied to determine MDA, based on 2-thiobarbituric acid (TBA) adduct formation. Each biological sample underwent a sample-specific preparation procedure. Hemolyzed erythrocytes were deproteinized with phosphoric acid and TBA solution [29–31]. Plasma and urine samples were mixed with 0.44 M phosphoric acid and 42 mM TBA solution [32]. Deproteinized biological samples were heated at 95°C for 60 min, chilled on ice, and centrifuged at 2500 ×g for 3 min at 4°C. Then, supernatants were filtered through a 0.45 *μ*m PTFE syringe filter (Woongki Science Co., Seoul, Korea). Creatinine-unadjusted urinary MDA concentration was used because the values of creatinine-adjusted, and -unadjusted MDA levels were highly correlated [33–35]. The mobile phase was 50 mM potassium phosphate buffer (pH 6.8) with methanol (7 : 3, v/v) for erythrocyte samples, and with methanol (8 : 2, v/v) for plasma and urine samples. Separation of MDA was performed on an HPLC (Shiseido Co. Ltd.) equipped with a 5 *μ*m Capcell Pak C18 column (4.6 mm id × 250 mm; Shiseido Co. Ltd.) with a flow rate of 1.0 mL/min at 40°C. The MDA was monitored with fluorescence detection (excitation *λ* = 527 nm and emission *λ* = 551 nm). The peak of the MDA–TBA adduct was calibrated with a 1,1,3,3-tetraethoxypropane solution [36].

### 2.6. Statistical Analysis

All analyses were two sided, with a *p* value < 0.05 indicating statistical significance. All statistical analyses were performed using the SAS software (version 9.4, SAS Institute Inc., Cary, NC, USA). Normal distribution and homogeneity of the data variance were tested by the Shapiro–Wilk's test. If not normally distributed, data were log-transformed before statistical analyses. Outliers were defined, and observations with absolute studentized residuals greater than three standard deviations were eliminated. Differences between the apparently healthy and oxidative stress condition groups were determined by the Student's *t*-test and chi-square test, for continuous and categorical variables, respectively. Multiple linear regression analyses were conducted to examine the association between RFS, plasma carotenoids, and MDA levels in plasma, erythrocytes, and urine, while adjusting for confounding variables (age, gender [37], and BMI [38]). For each predictor variable, the model provides a *β* coefficient and a *p* value that indicates the significance of the contribution of the variable towards the variability of plasma carotenoids or MDA levels in biological samples. We used a general linear model to test interactions of the groups with oxidative stress-related parameters (RFS, plasma carotenoids, and MDA in biological samples) with adjustment for confounding variables.

## 3. Results

### 3.1. Characteristics of Study Participants

First of all, we performed statistical tests to identify any significant differences between the healthy individuals and those presenting oxidative stress conditions. A total of 1229 participants (*n* = 823 apparently healthy and *n* = 406 oxidative stress conditions) were recruited into the cross-sectional study. Compared with the apparently healthy group, the oxidative stress condition group was significantly older and included more men (Table 1). The oxidative stress condition group had significantly higher anthropometric measures for systolic and diastolic blood pressure, fasting glucose, and triglyceride levels, but had lower high-density lipoprotein cholesterol compared to the apparently healthy group (*p* < 0.05 for all). Meanwhile, current smoking, low/medium education, and married adults were more present in the oxidative stress condition group (*p* < 0.05 for all) than the healthy cohort.

**Figure 2:**
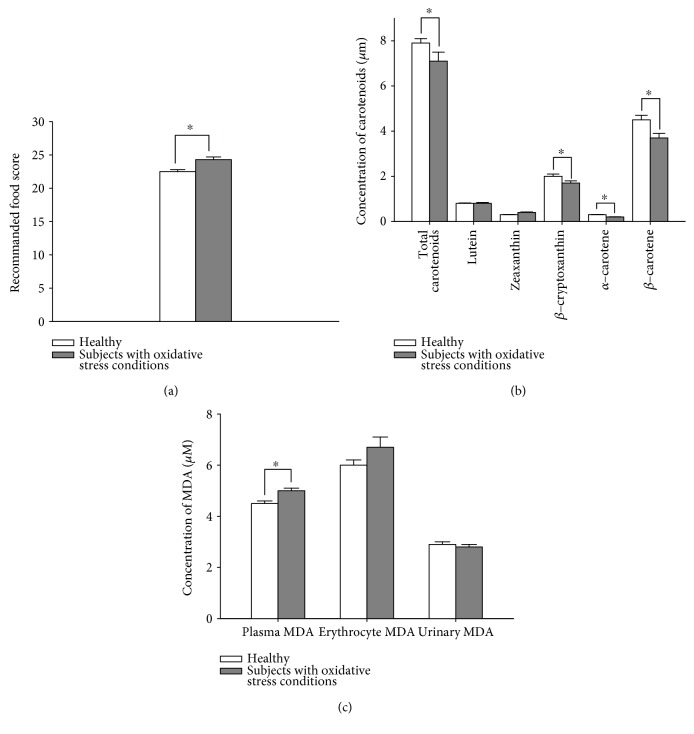
Characteristics of oxidative stress-related parameters in apparently healthy subjects and those with oxidative stress conditions: (a) recommended food score, (b) plasma concentration of carotenoids, and (c) malondialdehyde (MDA) levels in plasma, erythrocytes, and urine. Results are shown as mean ± standard error. ^∗^*p* < 0.005, significant difference between the apparently healthy and oxidative stress condition groups.

Oxidative stress-related parameters in the apparently healthy and oxidative stress condition groups are listed in Figure 2. The mean value of RFS and erythrocyte MDA was higher in the oxidative stress condition group than the apparently healthy group (*p* ≤ 0.001 for all), indicating better diet quality but higher oxidative stress in subjects with oxidative stress conditions. The apparently healthy group had a relatively higher concentration of total carotenoids, *β*-cryptoxanthin, *α*-carotene, and *β*-carotene (*p* ≤ 0.05 for all), which are indicative of lower oxidative stress.

However, although the oxidative stress condition group seemed to consume more carotenoids, these compounds might be used as antioxidants for quenching or removing ROS/RNS, due to high oxidative stress.

### 3.2. Association between Oxidative Stress-Related Parameters

Associations between RFS and plasma carotenoids resulting from multiple linear regression analyses are presented in Table 2. After adjusting for potential confounders, RFS was positively and significantly associated with total carotenoids (*β* = 0.007 per 1 score increment for the healthy group and 0.006 for the oxidative stress condition group) and *β*-carotene (*β* = 0.007 per 1 score increment for both groups), regardless of healthy state (*p* < 0.05 for all). This finding was expected, as higher plasma carotenoid levels reflect a better diet quality.

**Figure 3:**
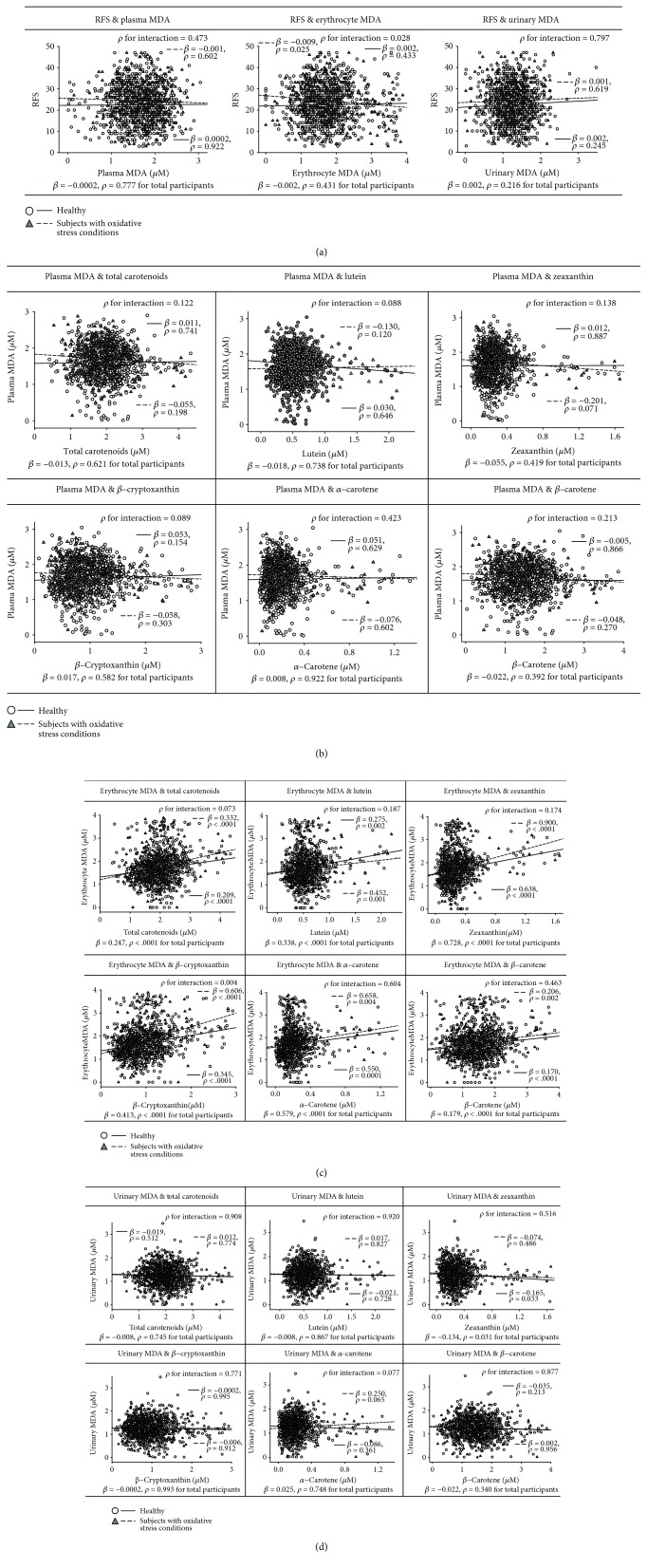
Association between oxidative stress-related parameters: (a) recommended food score (RFS) and malondialdehyde (MDA) in biological samples, (b) plasma MDA and plasma carotenoids, (c) erythrocyte MDA and plasma carotenoids, and (d) urinary MDA and plasma carotenoids. All analyses were adjusted for age, gender, body mass index, and physical activity. RFS, carotenoids, and MDA were considered to be representative of antioxidant-rich food consumption, blood antioxidants, and oxidative damage. The circle with dashed line and diamond with dotted line indicate the regression line and probability value for slope in the apparently healthy and oxidative stress condition groups, respectively. Difference among slopes and probability values for interaction (*p* for interaction) were analyzed between the two groups and for the effect of RFS and carotenoids on MDA by using the general linear model.

Associations of MDA in biological samples with RFS and plasma carotenoids resulting from multiple linear regression analyses are presented in Figure 3. No significant associations of plasma MDA were observed with RFS and plasma carotenoids in the two groups. There was a significantly negative association between RFS and erythrocyte MDA in the oxidative stress condition group (*β* = −0.009 per 1 score increment), but not in the apparently healthy group. The association between erythrocyte MDA and total and individual plasma carotenoids had a significant positive association in both the apparently healthy and oxidative stress condition groups (*p* < 0.005 for all carotenoids). The result above suggests that a better RFS reduced the oxidation status in the oxidative stress condition at the most active sites (erythrocytes) for oxidation, accomplished by the circulating carotenoids. For urinary MDA, a significant negative association with plasma zeaxanthin was only observed in apparently healthy subjects (*β* = −0.165 per 1-log unit increase, *p* = 0.033). Among the total participants, the increase of per 1-log unit of zeaxanthin led to a significant decrease in urinary MDA (*β* = −0.134, *p* = 0.031). These results might also be expected since zeaxanthin may have a potential role as a circulating antioxidant.

## 4. Discussion

In the current study, different patterns of association were found between RFS, plasma carotenoids, and MDA in biological samples according to the presence of oxidative stress conditions in the Korean adult population. The results were analyzed to better understand the link between antioxidant-rich food consumption, blood antioxidants, and oxidative damage.

The limitations of this study should be considered in interpreting the findings. First is the insufficiency of data on the other various oxidative stress markers. Individual markers of oxidative stress and antioxidants cannot and do not fully reflect the ongoing and complex biological process of redox homeostasis. MDA indicates oxidative lipid damage but is only one product of lipid peroxidation. Peripheral markers of oxidative damage to proteins and DNA are not available in this study. In the present study, erythrocyte MDA had a strong positive association with carotenoids. Erythrocytes contain high amounts of antioxidants, but their membrane can be easily affected by oxidative stress. The first line of defense against oxidative stress may be glutathione and endogenous antioxidant enzymes, such as superoxide dismutase and glutathione peroxidase [39, 40]. Thus, antioxidant defense mechanisms in erythrocytes require further investigation to exploit these outcomes. The next limitation is that as usual dietary intake data was not available, the analysis could not be adjusted for dietary energy or nutrient intake. In this context, the association of the mentioned oxidative stress-related parameters should be interpreted with caution, in the absence of detailed information about the nutritional content of the subject's habitual diet. However, self-assessment of dietary intake remains challenging owing to difficulties in recalling all food eaten, the ingredient composition of a recipe, or portion size. Plasma carotenoid concentrations have been considered to provide a more precise measurement for predicting dietary intake during absorption, metabolism, distribution, and excretion and give the least errors than self-reported dietary tests [25, 41]. However, these levels are still heavily affected by quenching oxidative stress and, thereby, may not be a reliable exposure biomarker. Lastly, the study has no experimental or longitudinal design, which is an inherent limitation of any retrospective case-control study, such as this one. The main strengths of this study are the relatively large sample size, the measurements of circulating carotenoids and MDA with gold-standard techniques, and determination of the temporal associations between diet quality, carotenoids, and MDA, according to health status.

A higher RFS can be considered to reflect a better antioxidant-rich food consumption, which is often shown to be represented in the plasma carotenoid levels. A statistically significant and consistent association was found between RFS and plasma carotenoids, especially total and *β*-carotene, whether in healthy individuals or subjects with oxidative stress conditions. It could be presumed that plasma total carotenoids and *β*-carotene, as dietary exposure biomarkers, reflected antioxidant-rich food consumption. It was attempted and proven that fruit, vegetable, and *β*-carotene intakes reported from diet quality indicators were significantly associated with plasma carotenoid concentrations (*α*-carotene, *β*-carotene, *β*-cryptoxanthin, and total) in Swedish men and women aged 50–64 yr (*n* = 1000) [42]. In addition, a higher diet quality score was significantly associated with total carotenoids (*α*-carotene, *β*-carotene, *β*-cryptoxanthin, lutein, zeaxanthin, and lycopene) in European adults (*n* = 1480) from the Food4ME study [43]. Therefore, plasma carotenoid levels may be considered as an objective and reliable assessment of food intake quality.

Erythrocytes are uniquely vulnerable to oxidative stress, considering the exposure to high concentrations of oxygen, resulting in high amounts of ROS [44, 45], which is increased by the inability to synthesize new protein and degradation of detoxifying enzymes as a result of the lack of nuclei and mitochondria [46]. It is also known that the erythrocyte membrane is prone to lipid peroxidation under oxidative stress that involves cleavage of polyunsaturated fatty acids at their double bonds, leading to the formation of MDA [39]. Subjects with oxidative stress conditions are thought to have increased oxidative stress, owing to decreased glutathione and membrane sulfhydryl groups and higher lipid peroxidation. It was proven that an antioxidant-rich diet might protect from oxidative damage in erythrocytes under oxidative stress conditions, such as *ex vivo* oxidative stress [46], smokers [47], and type 2 diabetic patients [48]. Fruit and vegetable consumption causes a significant increase in plasma antioxidant concentrations (*α*-tocopherol, ascorbic acid, and *β*-carotene), thereby contributing to the strengthening of the defense mechanisms against excessive oxidative stress by supporting the endogenous antioxidant activity [40, 49, 50] and by a synergistic action and interaction between different antioxidants [49]. A novel finding in the current study is that a robust positive association between erythrocyte MDA and plasma carotenoids was found, regardless of the health status. Due to the increased vulnerability of erythrocytes to oxidative stress [44, 45], a high antioxidant requirement is expected for these cells, which is proposed to result in a positive relationship between erythrocyte MDA and plasma carotenoid levels.

Antioxidant-rich food consumption or blood antioxidants were further associated with improved oxidative status, but the pattern is slightly different regarding health conditions. Plasma zeaxanthin appeared to be negatively associated with urinary MDA in healthy subjects. Since healthy subjects are presumed to have a better antioxidant status than their oxidative stress counterparts, high antioxidant capacity and decreased oxidative damage could be observed. However, the depletion of carotenoids may be responsible for the lack of this association in the oxidative stress condition group. The antioxidant mechanism of carotenoids is the ability to effectively quench and neutralize highly active radical forms of oxygen, as well as free radicals [51]. It has also been suggested that carotenoids can prevent many degenerative diseases, including cardiovascular diseases, neurological diseases, diet-related cancer, and age-related macular degeneration, via prevention of lipid peroxidation [51, 52]. As lipophilic antioxidants and the inhibitors of lipoxygenase, carotenoids are incorporated into lipid membranes where they act to suppress free radical chain reactions (propagation of lipid peroxidation) and protect polyunsaturated fatty acids from oxidative damages [51].

## 5. Conclusions

In conclusion, this study demonstrates that plasma antioxidants reflect diet quality. Moreover, the association between carotenoids and MDA differs according to the health status of the participants. Quenching of the oxidative response in the erythrocytes leads to a reduction of plasma carotenoid levels. The combination of RFS, plasma carotenoids, and erythrocyte MDA can be used to monitor oxidative status more effectively than the individual biomarkers. Further studies need to be done to determine whether (1) RFS and plasma carotenoids at baseline would have a predictive relationship with oxidative stress conditions using longitudinal cohort data and (2) whether erythrocytes would be an important compartment for antioxidant defense mechanisms, by analyzing endogenous molecules.

## Figures and Tables

**Table 1:** General characteristics of the participants^1^.

	Total (*n* = 1229)	Apparently healthy (*n* = 823)	Oxidative stress condition (*n* = 406)	*p* value^2^
Age (yr)	47.4 ± 11.3	45.3 ± 10.7	51.8 ± 11.2	<0.0001
Gender (%)				<0.0001
Men	751 (61.1)	446 (54.2)	377 (75.1)	
Women	478 (38.9)	305 (45.8)	101 (24.9)	
Anthropometric measures				
Body mass index (kg/m^2^)	23.9 ± 3.6	22.6 ± 2.4	26.4 ± 4.1	<0.0001
Waist circumference (cm)	84.4 ± 9.1	81.0 ± 7.1	91.1 ± 8.9	<0.0001
Waist-to-hip ratio	0.9 ± 0.1	0.9 ± 0.1	0.9 ± 0.1	<0.0001
Body fat percentage (%)	27.3 ± 6.8	26.1 ± 6.5	29.7 ± 6.8	<0.0001
Blood pressure (mmHg)				
Systolic	119.2 ± 14.2	115.4 ± 13.2	126.8 ± 13.2	<0.0001
Diastolic	81.0 ± 10.2	79.0 ± 9.4	85.1 ± 10.5	<0.0001
Biochemistry (mg/dL)				
Fasting glucose	94.4 ± 18.3	90.4 ± 15.3	102.5 ± 21.0	<0.0001
Cholesterol	195.4 ± 35.4	195.3 ± 33.6	195.6 ± 38.9	0.774
High-density lipoprotein cholesterol	54.0 ± 13.1	57.1 ± 13.0	47.6 ± 10.9	<0.0001
Low-density lipoprotein cholesterol	119.0 ± 32.7	119.4 ± 31.6	118.0 ± 35.0	0.243
Triglycerides	112.6 ± 66.0	94.1 ± 51.7	150.0 ± 75.3	<0.0001
Physical activity (METs^3^-min/wk)	221.5 ± 344.5	215.1 ± 332.4	234.5 ± 367.7	0.214
Lifestyle factors				
Marital status, married (%)	994 (81.6)	649 (79.4)	345 (86.0)	0.005
Education ≥ 12 yr (%)	919 (75.3)	647 (53.1)	272 (22.3)	<0.0001
Current smoking (%)	232 (18.9)	127 (15.4)	105 (25.9)	<0.0001

^1^Values are mean ± standard deviation or number of subjects (%). ^2^Differences between the two groups were assessed by a *t*-test analysis for continuous variables and chi-square tests for categorical variables. ^3^Metabolic equivalent of tasks.

**Table 2:** Associations between recommended food score and plasma concentrations of carotenoids.^1^

	Recommended food score			
	Model 1^2^		Model 2^3^	
	Estimate	*p* value	Estimate	*p* value
Total				
Carotenoids				
Total	0.007	<0.0001	0.006	<0.0001
Lutein	0.003	<0.0001	0.002	0.042
Zeaxanthin	0.001	0.116	0.001	0.275
*β*-Cryptoxanthin	0.003	0.018	0.003	0.020
*α*-Carotene	0.001	0.017	0.001	0.011
*β*-Carotene	0.007	<0.0001	0.007	<0.0001
Apparently healthy				
Carotenoids				
Total	0.008	<0.0001	0.007	0.003
Lutein	0.003	0.002	0.002	0.046
Zeaxanthin	0.001	0.070	0.001	0.145
*β*-Cryptoxanthin	0.004	0.032	0.003	0.074
*α*-Carotene	0.001	0.025	0.001	0.041
*β*-Carotene	0.008	<0.0001	0.007	0.001
Oxidative stress condition				
Carotenoids				
Total	0.008	0.014	0.006	0.037
Lutein	0.002	0.125	0.001	0.447
Zeaxanthin	0.0001	0.911	−0.0001	0.931
*β*-Cryptoxanthin	0.004	0.068	0.004	0.115
*α*-Carotene	0.001	0.128	0.001	0.136
*β*-Carotene	0.008	0.008	0.007	0.018

^1^Carotenoids were log_10_-transformed before analysis. Data were expressed as regression coefficients (parameter estimate) and *p* value. ^2^Unadjusted. ^3^Adjusted for age, gender, body mass index, and physical activity.

## Data Availability

The data used to support the findings of this study are available from the corresponding author upon request.

## References

[1] Roberts C. K., Sindhu K. K. (2009). Oxidative stress and metabolic syndrome. *Life Sciences*.

[2] Furukawa S., Fujita T., Shimabukuro M. (2004). Increased oxidative stress in obesity and its impact on metabolic syndrome. *The Journal of Clinical Investigation*.

[3] Maritim A. C., Sanders A., Watkins J. B. (2003). Diabetes, oxidative stress, and antioxidants: a review. *Journal of Biochemical and Molecular Toxicology*.

[4] Monsen E. R. (2000). Dietary reference intakes for the antioxidant nutrients: vitamin C, vitamin E, selenium, and carotenoids. *Journal of the Academy of Nutrition and Dietetics*.

[5] Key T. J., Allen N. E., Spencer E. A., Travis R. C. (2002). The effect of diet on risk of cancer. *The Lancet*.

[6] Amine E. K., Baba N. H., Belhadj M. (2003). *Diet, nutrition and the prevention of chronic diseases*.

[7] Holt E. M., Steffen L. M., Moran A. (2009). Fruit and vegetable consumption and its relation to markers of inflammation and oxidative stress in adolescents. *Journal of the American Dietetic Association*.

[8] Ristow M., Zarse K., Oberbach A. (2009). Antioxidants prevent health-promoting effects of physical exercise in humans. *Proceedings of the National Academy of Sciences*.

[9] Persson C., Sasazuki S., Inoue M. (2008). Plasma levels of carotenoids, retinol and tocopherol and the risk of gastric cancer in Japan: a nested case–control study. *Carcinogenesis*.

[10] Lidebjer C., Leanderson P., Ernerudh J., Jonasson L. (2007). Low plasma levels of oxygenated carotenoids in patients with coronary artery disease. *Nutrition, Metabolism and Cardiovascular Diseases*.

[11] Gura K. M., Mulberg A. E., Mitchell P. D. (2018). Pediatric intestinal failure-associated liver disease: challenges in identifying clinically relevant biomarkers. *Journal of Parenteral and Enteral Nutrition*.

[12] Calder P. C., Boobis A., Braun D. (2017). Improving selection of markers in nutrition research: evaluation of the criteria proposed by the ILSI Europe Marker Validation Initiative. *Nutrition Research Reviews*.

[13] Betteridge D. J. (2000). What is oxidative stress?. *Metabolism*.

[14] Nielsen F., Mikkelsen B. B., Nielsen J. B., Andersen H. R., Grandjean P. (1997). Plasma malondialdehyde as biomarker for oxidative stress: reference interval and effects of life-style factors. *Clinical Chemistry*.

[15] Kim J. Y., Yang Y. J., Yang Y. K. (2011). Diet quality scores and oxidative stress in Korean adults. *European Journal of Clinical Nutrition*.

[16] Couillard C., Lemieux S., Vohl M.-C., Couture P., Lamarche B. (2016). Carotenoids as biomarkers of fruit and vegetable intake in men and women. *British Journal of Nutrition*.

[17] Valko M., Leibfritz D., Moncol J., Cronin M. T., Mazur M., Telser J. (2007). Free radicals and antioxidants in normal physiological functions and human disease. *The International Journal of Biochemistry & Cell Biology*.

[18] Watters J. L., Satia J. A., da Costa K. A. (2009). Comparison of three oxidative stress biomarkers in a sample of healthy adults. *Biomarkers*.

[19] Matsuda M., Shimomura I. (2013). Increased oxidative stress in obesity: implications for metabolic syndrome, diabetes, hypertension, dyslipidemia, atherosclerosis, and cancer. *Obesity research & clinical practice*.

[20] Bloomer R. J. (2007). Decreased blood antioxidant capacity and increased lipid peroxidation in young cigarette smokers compared to nonsmokers: impact of dietary intake. *Nutrition Journal*.

[21] Cherubini A., Ruggiero C., Polidori M. C., Mecocci P. (2005). Potential markers of oxidative stress in stroke. *Free Radical Biology and Medicine*.

[22] World Health Organization (2003). *Diet, Nutrition, and the Prevention of Chronic Diseases: Report of a Joint WHO/FAO Expert Consultation*.

[23] World Health Organization (2008). *Waist circumference and waist-hip ratio: report of a WHO expert consultation*.

[24] Oh J. Y., Yang Y. J., Kim B. S., Kang J. H. (2007). Validity and reliability of Korean version of International Physical Activity Questionnaire (IPAQ) short form. *Korean Journal of Family Medicine*.

[25] Carlsen M. H., Karlsen A., Lillegaard I. T. L. (2011). Relative validity of fruit and vegetable intake estimated from an FFQ, using carotenoid and flavonoid biomarkers and the method of triads. *British Journal of Nutrition*.

[26] Lykkesfeldt J. (2001). Determination of malondialdehyde as dithiobarbituric acid adduct in biological samples by HPLC with fluorescence detection: comparison with ultraviolet-visible spectrophotometry. *Clinical Chemistry*.

[27] Kant A. K., Schatzkin A., Graubard B. I., Schairer C. (2000). A prospective study of diet quality and mortality in women. *JAMA*.

[28] Kauffman L. D., Sokol R. J., Jones R. H., Awad J. A., Rewers M. J., Norris J. M. (2003). Urinary F2-isoprostanes in young healthy children at risk for type 1 diabetes mellitus. *Free Radical Biology & Medicine*.

[29] Arsova-Sarafinovska Z., Aydin A., Sayal A. (2007). Rapid and simple determination of plasma and erythrocyte MDA levels in prostate cancer patients by a validated HPLC method. *Journal of Liquid Chromatography & Related Technologies*.

[30] Mawatari S., Saito K., Murakami K., Fujino T. (2004). Absence of correlation between glycated hemoglobin and lipid composition of erythrocyte membrane in type 2 diabetic patients. *Metabolism-Clinical and Experimental*.

[31] Cipierre C., Haÿs S., Maucort-Boulch D., Steghens J.-P., Picaud J.-C. (2013). Malondialdehyde adduct to hemoglobin: a new marker of oxidative stress suitable for full-term and preterm neonates. *Oxidative Medicine and Cellular Longevity*.

[32] Suttnar J., Čermák J., Dyr J. E. (1997). Solid-phase extraction in malondialdehyde analysis. *Analytical Biochemistry*.

[33] Yoon H.-S., Lee K.-M., Lee K.-H., Kim S., Choi K., Kang D. (2012). Polycyclic aromatic hydrocarbon (1-OHPG and 2-naphthol) and oxidative stress (malondialdehyde) biomarkers in urine among Korean adults and children. *International Journal of Hygiene and Environmental Health*.

[34] Lee K.-H., Vermeulen R., Lenters V., Cho S.-H., Strickland P. T., Kang D. (2009). Determinants of urinary 1-hydroxypyrene glucuronide in South Korean children. *International Archives of Occupational and Environmental Health*.

[35] Kim B., Kwon B., Jang S., Kim P.-G., Ji K. (2016). Major benzophenone concentrations and influence of food consumption among the general population in Korea, and the association with oxidative stress biomarker. *Science of the Total Environment*.

[36] Khoschsorur G. A., Winklhofer-Roob B. M., Rabl H., Auer T., Peng Z., Schaur R. J. (2000). Evaluation of a sensitive HPLC method for the determination of malondialdehyde, and application of the method to different biological materials. *Chromatographia*.

[37] Black C. N., Bot M., Scheffer P. G., Penninx B. W. J. H. (2016). Sociodemographic and lifestyle determinants of plasma oxidative stress markers 8-OHdG and F2-isoprostanes and associations with metabolic syndrome. *Oxidative Medicine and Cellular Longevity*.

[38] Hughes K. J., Mayne S. T., Blumberg J. B., Ribaya-Mercado J. D., Johnson E. J., Cartmel B. (2009). Plasma carotenoids and biomarkers of oxidative stress in patients with prior head and neck cancer. *Biomarker insights*.

[39] Rizvi S. I., Maurya P. K. (2007). Markers of oxidative stress in erythrocytes during aging in humans. *Annals of the New York Academy of Sciences*.

[40] Chiste R. C., Freitas M., Mercadante A. Z., Fernandes E. (2014). Carotenoids inhibit lipid peroxidation and hemoglobin oxidation, but not the depletion of glutathione induced by ROS in human erythrocytes. *Life Sciences*.

[41] Potischman N. (2003). Biologic and methodologic issues for nutritional biomarkers. *The Journal of Nutrition*.

[42] Nybacka S., Lindroos A. K., Wirfält E. (2016). Carotenoids and alkylresorcinols as objective biomarkers of diet quality when assessing the validity of a web-based food record tool and a food frequency questionnaire in a middle-aged population. *BMC Nutrition*.

[43] Fallaize R., Livingstone K. M., Celis-Morales C. (2018). Association between diet-quality scores, adiposity, total cholesterol and markers of nutritional status in European adults: findings from the Food4Me study. *Nutrients*.

[44] Kumaraguruparan R., Subapriya R., Kabalimoorthy J., Nagini S. (2002). Antioxidant profile in the circulation of patients with fibroadenoma and adenocarcinoma of the breast. *Clinical Biochemistry*.

[45] Ripple M., Mulcahy R. T., Wilding G. (1993). Characteristics of the glutathione/glutathione-S-transferase detoxification system in melphalan resistant human prostate cancer cells. *The Journal of Urology*.

[46] Pandey K. B., Rizvi S. I. (2009). Protective effect of resveratrol on formation of membrane protein carbonyls and lipid peroxidation in erythrocytes subjected to oxidative stress. *Applied Physiology, Nutrition, and Metabolism*.

[47] Brown K. M., Morrice P. C., Duthie G. G. (1997). Erythrocyte vitamin E and plasma ascorbate concentrations in relation to erythrocyte peroxidation in smokers and nonsmokers: dose response to vitamin E supplementation. *The American Journal of Clinical Nutrition*.

[48] Rizvi S. I., Zaid M. A., Anis R., Mishra N. (2005). Protective role of tea catechins against oxidation-induced damage of type 2 diabetic erythrocytes. *Clinical and Experimental Pharmacology and Physiology*.

[49] Harasym J., Oledzki R. (2014). Effect of fruit and vegetable antioxidants on total antioxidant capacity of blood plasma. *Nutrition*.

[50] Tesoriere L., Butera D., Pintaudi A. M., Allegra M., Livrea M. A. (2004). Supplementation with cactus pear (*Opuntia ficus-indica*) fruit decreases oxidative stress in healthy humans: a comparative study with vitamin C. *The American Journal of Clinical Nutrition*.

[51] Bouayed J., Bohn T. (2012). Dietary derived antioxidants: implications on health. *Nutrition, Well-Being and Health*.

[52] Goupy P., Carail M., Giuliani A., Duflot D., Dangles O., Caris-Veyrat C. (2018). Carotenoids: experimental ionization energies and capacity at inhibiting lipid peroxidation in a chemical model of dietary oxidative stress. *The Journal of Physical Chemistry B*.

